# Anterior Segment Eye Diseases and Quality of Life: An Updated Comprehensive Narrative Review

**DOI:** 10.18502/jovr.v21.18248

**Published:** 2026-05-24

**Authors:** Masoud Khorrami-Nejad, Foroozan Narooie-Noori, Qasim Al-khafaji, Hesam Hashemian, Sorcha Ní Dhubhghaill, Sheraz Daya

**Affiliations:** ^1^School of Rehabilitation, Tehran University of Medical Sciences, Tehran, Iran; ^2^Translational Ophthalmology Research Center, Farabi Eye Hospital, Tehran University of Medical Sciences, Tehran, Iran; ^3^Students' Scientific Research Center, Tehran University of Medical Sciences, Tehran, Iran.; ^4^Faculty of Medicine and Health Sciences, Department of Ophthalmology, Visual Optics and Visual Rehabilitation, University of Antwerp, Antwerp, Belgium; ^5^Department of Ophthalmology, Antwerp University Hospital, Antwerp, Belgium; ^6^Centre for Sight, East Grinstead, West Sussex, UK

**Keywords:** Anterior Segment, Dry Eye Disease, Keratoconus, Quality of Life, Strabismus

## Abstract

Anterior segment eye diseases significantly affect patients' quality of life (QoL) by impairing physical health, psychological well-being, social interactions, and economic stability. Conditions such as dry eye disease, blepharitis, conjunctivitis, keratoconus, keratitis, and corneal dystrophies cause chronic discomfort, visual disturbance, and emotional distress, leading to reduced daily functioning and work productivity. Other disorders like chalazion, trichiasis, ptosis, strabismus, and thyroid eye disease entail a psychological burden by affecting appearance and social confidence. This narrative review comprehensively examines the multidimensional impact of anterior segment diseases on QoL. A systematic literature search was conducted in PubMed, Scopus, Web of Science, and Google Scholar for English-language articles published between December 1997 and February 2025. Using relevant keywords and Boolean operators, we initially identified 854 articles. Following duplicate removal and screening based on inclusion and exclusion criteria focused on QoL-related outcomes in anterior segment diseases, we selected 72 high-quality studies for the review. This methodology ensured a thorough and focused analysis of the available evidence. Findings highlight the importance of addressing both clinical symptoms and psychosocial effects in management strategies. Future research should emphasize interventions that improve ocular function alongside overall well-being.

##  INTRODUCTION

According to the World Health Organization, quality of life (QoL) is a broad concept related to an individual's overall well-being. It consists of multiple dimensions, covering physical health, psychological state, degree of independence, social relationships, personal beliefs, and relationship to salient features of the environment.^[1]^ The anterior segment of the eye plays a crucial role in maintaining visual function, ocular health, and overall QoL. Diseases affecting this region, including dry eye disease (DED), blepharitis, conjunctivitis, keratoconus (KCN), and corneal dystrophies, can lead to significant discomfort, impaired vision, and long-term functional limitations.^[2,3,4,5,6,7,8,9,10,11,12,13]^ These conditions not only affect visual acuity but also contribute to chronic pain, psychological distress, and social withdrawal.

Beyond the physical symptoms, anterior segment diseases have a profound impact on mental health and economic stability. Many patients experience anxiety, depression, and reduced self-esteem due to persistent visual challenges and aesthetic concerns.^[6,14]^ The economic burden of these conditions is also substantial, with direct costs related to medical treatments and indirect costs arising from work absenteeism and reduced productivity. The social implications are equally severe, as individuals with anterior segment disorders often struggle with diminished independence and decreased participation in professional and leisure activities.

Given the widespread prevalence and multifaceted impact of these conditions, it is essential to understand how anterior segment diseases influence QoL. This review aims to summarize the effects of various anterior segment diseases on patients' QoL and evaluate their physical, psychological, social, and economic consequences. Additionally, it discusses current management strategies and potential interventions to improve patient outcomes.

##  METHODS

The literature for this review was gathered through a comprehensive search of peer-reviewed articles, clinical studies, and relevant publications focusing on anterior segment diseases and their impact on QoL. The databases searched included PubMed, Scopus, Web of Science, and Google Scholar. The search covered articles published in English from December 1997 up to February 2025. A combination of keywords and Boolean operators was used to refine the search strategy. Keywords included “anterior segment diseases”, “quality of life”, “dry eye disease”, “keratoconus”, “blepharitis”, “conjunctivitis”, “keratitis”, “corneal dystrophies”, “glaucoma”, and “cataract”. Initially, a total of 854 articles were retrieved based on title and abstract screening. After duplicates (*n* = 244) were removed, 610 articles remained. These articles were screened for relevance to our topic based on title and abstract. Our exclusion criteria were a lack of QoL-related data, a lack of focus on anterior eye segment disorders, and non-English articles. Therefore, 360 articles were further excluded based on the specified criteria. Inclusion criteria comprised studies focused on anterior segment diseases, the use of validated QoL assessment instruments or outcomes, and clear methodology and study design publication in English. This process resulted in 101 full-text articles, of which 72 met all criteria and were included in the final analysis.

This protocol ensured that only high-quality and directly relevant studies were analyzed, contributing to a comprehensive and reliable review of the topic.

### Dry Eye Disease (DED) and Quality of Life (QoL)

This subsection summarizes findings from 18 studies focusing on the impact of DED on various domains of QoL, including physical health, psychological well-being, social functioning, and economic burden.

DED refers to a multifactorial ocular surface and tear disorder with symptoms of discomfort, visual disturbance, tear film instability, and often inflammation and damage of the ocular surface.^[14,15]^ DED is a common condition affecting millions of people globally, with reported prevalence rates varying from 5% to 50% depending on the study population and diagnostic criteria used. In the US, DED affects an estimated 5 million people aged 50 years and older.^[16,17,18,19]^ It is more prevalent among females, especially postmenopausal females, and is related to risk factors like aging, autoimmune disorders (e.g., Sjögren syndrome), contact lens use, and other environmental factors like low humidity.^[2]^

DED strongly affects patients' QoL and impacts physical, emotional, and social functioning as well as the ability to perform daily activities.^[3,20]^ Studies on the association of DED and QoL demonstrate the heavy burden of this disease, extending beyond visual impairment to encompass mental health aspects in addition to reduced work productivity.^[21]^ These significant physical and psychological burdens are due to the chronic nature of the disease and the persistence of symptoms such as dryness, burning, stinging, and fluctuating vision. These symptoms interfere with daily living activities and significantly reduce the patients' ability to function personally and professionally.^[3,22]^

DED has far-reaching consequences affecting various QoL domains.^[3,20]^ Patient-reported outcomes and validated questionnaires corroborate that DED negatively impacts multiple aspects of these domains.

#### Physical health

Patients with DED frequently present with chronic pain and discomfort, symptoms of grittiness, dryness, and burning. These symptoms can cause ocular fatigue and contribute to challenges in performing vision-dependent tasks such as reading, driving, and using digital devices.^[23]^ Furthermore, reduced or fluctuating vision, particularly with prolonged viewing, is strongly associated with diminished functional visual acuity—a measure of vision quality that better reflects its use in activities of daily living.^[3]^

#### Psychological well-being

The psychological impact of DED can be debilitating, with many patients experiencing more anxiety, depression, and emotional distress than those without this condition.^[24]^ DED symptoms can be chronic and frequently unremitting, interfering with daily functioning and leading to frustration, sadness, and withdrawal from social activities. Indeed, patients with DED, especially those with moderate to severe symptoms, are known to score lower in generic QoL questionnaires such as mental health domains of the Short Form-36 (SF-36).^[3]^

#### Social life and occupation

Patients with DED may have difficulty with leisure activities, including watching television or driving at night, due to visual disturbances and light sensitivity.^[2]^ These limitations may result in decreased independence and involvement in social activities. DED also affects productivity in the workplace. Presenteeism (i.e., the act of working with mild/moderate symptoms or at lower productivity levels) is a large part of DED's impact on individuals and results in considerable economic loss. Reddy et al found that patients with DED miss 2-5 days of work each year due to their symptoms, and presenteeism accounts for 191-208 days of reduced productivity annually.^[25]^ This underscores the economic burden of the disease, which is much broader than health expenses.

#### Economic burden

The economic burden of DED is high, comprising direct costs (e.g., physician office visits and medical treatments) and indirect costs (e.g., productivity loss). A study in the United States estimated the annual direct cost of managing DED at $783 per patient, with a total economic burden of $3.84 billion for the healthcare system. The indirect costs were mainly related to reduced workplace productivity and were estimated at $55.4 billion per year.^[26]^

#### Measuring QoL in DED

Many disease-specific QoL questionnaires have been developed to understand and address the impact of DED.

• Ocular Surface Disease Index (OSDI): A 12-item questionnaire that is widely used to evaluate symptoms, vision-related functioning, and environmental triggers. It is a validated measure that can differentiate between mild, moderate, and severe DED.^[23]^
• Impact of Dry Eye on Everyday Life (IDEEL): A 57-item questionnaire that covers symptom bother, effects on daily life (i.e., emotional well-being, activity limitations), and treatment satisfaction. It offers an in-depth assessment of the impact of the disease on patients' QoL; however, compared with other commonly used dry eye questionnaires, it is more time-consuming to administer.^[24]^
• National Eye Institute Visual Function Questionnaire (NEI VFQ-25): A general vision-related QoL tool used in studies about the DED impact on general aspects of visual functioning and mental health. It also includes subscales for ocular pain, general vision, and social functioning.^[3]^

Without these tools, clinicians cannot evaluate DED severity and its impact on a patient's life, nor can they measure treatment efficacy. Table 1 reports the health-related QoL outcomes in patients with the most common anterior segment diseases.

**Table 1:** Health-related quality of life outcomes in patients with the most common anterior segment diseases

**Diseases**	**Country**	**Reference**	**Study design**	**QoL instruments**	**Sample size (Age of participants)**	**Main Findings**
Dry Eye Disease	USA	Nichols et al^[27]^	Prospective observational study	> NEI-VFQ-25	75 adults with DED (21.4 to 81.0Median: 46.2)	- Patients with dry eye exhibit reduced scores on the ocular pain subscale.
	USA	Schiffman et al^[28]^	Cross-sectional observational study	Short Form-36 Health Survey > NEI VFQ-25 Ocular Surface Disease Index Survey	56 adults with DED(13.9 ± 52.7)	- Utilities for severe dry eye are comparable to those of conditions like class III/IV angina (0.71), which are well-known for reducing health utilities.
	USA	Rajagopalan et al^[29]^	Cross-sectional comparative study	> SF-36 > EQ-5D > IDEEL	130 non-SS KCS (55.18 ± 15.26) 32 with SS(58.25 ± 11.78) 48 controls (39.23 ± 11.76)	- Significant differences across severity levels were observed in most SF-36 scales, all EQ-5D scales, and all IDEEL scales, except for Treatment Satisfaction. - Regardless of the severity criteria applied, IDEEL scales consistently outperformed the generic QoL measures. - While most SF-36 scales outperformed the EQ-5D QoL scale, the EQ-5D visual analog scale performed better than the SF-36 scales, except for General Health Perceptions.
	USA	Miljanović et al^[30]^	Cross-sectional comparative study	> DES questionnaire	690 adults with DED (39 to 90 years)	- Individuals with DES were more likely to report difficulties with reading, performing professional tasks, using a computer, watching TV, and driving both during the day and at night.
	France	Baudouin et al^[31]^	Cross-sectional study	> OSD-QoL	214 adults with DED (45 to 76)	- The mean scores for all OSD-QoL dimensions indicated impairment, with notable impacts on “Acknowledgement of the disease,” “Giving up makeup,” and “Fear for the future.”
	China	Labbé et al^[32]^	Population-based cross-sectional study	Zung self-rated depression scale	1456 adults (both control and DED groups)(64.82 ± 9.47)	- Definite depression was more common in patients with DED compared to those without DED. - Depression scores were found to correlate with the severity of dry eye symptoms.
Cataract	Finland	Uusitalo et al^[33]^	Cross-sectional study	> VF-14 > VF-7	142 adults with first eye surgery(N/A in the article)	- Seven items from the VF-14 that best correlated with patient satisfaction were selected for inclusion in the VF-7. - The items, ranked from best to worst correlation, were: nighttime driving, reading small print, watching television, seeing steps/stairs/curbs, reading traffic/street/store signs, cooking, and doing fine handwork.
	UK	Chak et al^[34]^	Cross-sectional study	> PedsQL 4.0 > Pediatric HRQOL	41 Children with congenital cataract(6 to 12 years)	- The mean physical health score self-reported by children was higher than the psychosocial health score. - There was considerable variation in the agreement of scores reported by individual child–parent pairs.
	Poland	Oleś et al^[35]^	Cross-sectional study	Life Quality Questionnaire > HRQOL	193 adults with cataract (66.47 ± 12.03)	- The greatest difficulties reported were with reading small print, newspapers, and prices, while patients coped relatively well with recognizing colors and food. - The most important changes after surgery were related to activities such as recognizing colors, food, and objects; cooking; housework; reading large-size letters; and recognizing money and faces.
	Vietnam	To et al^[36]^	Prospective cohort study	> NEI VFQ-25	413 adults with cataract, 247 completed the follow-up assessment after cataract surgery (50 to 70+)	- Vision-related Quality of Life (VRQoL) significantly improved after cataract surgery, particularly after surgeries on both eyes. - Binocular contrast sensitivity and stereopsis were associated with changes in VRQoL following cataract surgery.
	Nigeria	Abdullahi et al^[37]^	Prospective cohort study	> VF-14 > QOL	200 adults with cataract (45 to 85 years)	- There was a positive correlation between visual acuity (VA) and subjective visual function, as well as between VA and QoL. - Functional vision improved after cataract surgery, with more remarkable improvements among blind patients, whose mean VF and QoL scores were 58.3 and 60.5, respectively.
	Jordan	Al Zubi et al^[38]^	Qualitative study	Semi-structured instrument with open-ended questions	100 adults with cataract (N/A in the article)	- Cataract surgery is effective in improving participants' QoL by providing psychological, social, and emotional support.
QoL, quality of life; NEI-VFQ-25, National Eye Institute Visual Functioning Questionnaire; DED, dry eye disease; SF-36, 36-Item Short Form Health Survey; EQ-5D, European Quality of Life Group 5 Dimensions; IDEEL, Impact of Dry Eye on Everyday Life; Non-SS KCS, Non-Sjogren's Keratoconjunctivitis Sicca; SS, Sjogren's Syndrome; DES, dry eye syndrome; OSDI-QoL, Ocular Surface Disease Index; VF-14, Visual Function Index; PedsQL, Pediatric Quality of Life.						

### Blepharitis and Quality of Life (QoL)

This subsection summarizes findings from eight studies examining the impact of blepharitis on QoL, focusing on physical symptoms, psychological effects, social functioning, and the role of effective management strategies.

#### Physical health

The chronic nature of the disease, together with associated symptoms, leads to an adverse effect on patients' QoL. The chronic symptoms, including itching, burning, soreness, and blurry vision, can disrupt daily activities such as reading, driving, and extended screen time. The recurrence of the condition and the inconvenience of ongoing management feed into this burden on patients, contributing to frustration and decreased overall well-being.^[5]^

#### Social life and occupation

In severe cases, eyelid swelling, scaling, and lash misdirection can cause disturbance of self-consciousness and social withdrawal, reducing job prospects. These visible symptoms are frequently a source of emotional distress and can lead to anxiety or depression in some affected individuals.^[6]^

#### Psychological well-being

The psychological impact of not achieving symptom relief despite receiving treatment is well-documented. This finding highlights the critical need to address the complex interplay between psychological and physical factors involved in disease management.^[5]^

#### Effective management

Effective management (through eyelid hygiene and proper pharmacological means) can help improve the clinical outcome and QoL of patients. Educating patients about the chronic nature of the disease and realistic treatment goals will help reduce frustration and improve their overall satisfaction with care.^[5]^

### Conjunctivitis and Quality of life (QoL)

This subsection is based on findings from nine studies examining the impact of conjunctivitis, particularly allergic conjunctivitis (AC), on QoL, covering physical symptoms, emotional and psychological effects, economic burden, and impact on patients and their families.

Conjunctivitis, especially AC, contributes to a considerable reduction in QoL for the patient, has a recurrent nature, and is associated with itching, redness, tearing, and photophobia. These symptoms commonly impact daily activities, work efficiency, and socialization, thereby placing a significant burden on the individual. A Portuguese study observed significant impairment in self-perceived health status among patients with AC experiencing acute episodes, with an EQ-5D visual analogue scale (VAS) score of 47 compared with 88.7 during asymptomatic periods.^[39]^ The economic and functional repercussions of conjunctivitis further exacerbate its impact on QoL. A substantial proportion of patients (16%) in the same study reported missing work or school due to ocular allergy, with a median absence of 2 days. This absenteeism reflects the direct and indirect costs associated with conjunctivitis and its management.^[39]^

#### Psychological well-being

A study conducted in China revealed that children with AC, particularly those with more severe forms like vernal keratoconjunctivitis (VKC) or atopic keratoconjunctivitis (AKC), experienced significant QoL reductions. The Pediatric Quality of Life Inventory (PedsQL) scores for these children were notably lower than those of healthy controls, with emotional functioning and school performance being the most negatively affected. The findings emphasize that persistent symptoms and chronic discomfort can lead to psychological stress and hinder educational performance.^[40]^ Parents of children with conjunctivitis also experience a decline in QoL, particularly due to concerns about their child's health and the recurring nature of the condition. In the same Chinese study, parents of children with VKC or AKC exhibited lower QoL scores, with “worry” being the most affected domain. This highlights the ripple effect of conjunctivitis, as it not only impacts patients but also imposes a psychological and emotional burden on their families.^[40]^

#### Economic burden

Seasonal allergic conjunctivitis (SAC) has a substantial effect on QoL and economic costs. In a Spanish study, QoL scores for vision, pain, mental health, and role limitations were significantly lower among sufferers, who rated their EQ-VAS health an average of 80.09 (vs. 83.34 in controls). The total annual cost per affected individual, including direct costs and loss of productivity, was estimated at €348.50, significantly higher than expected amounts in the UK, mainly owing to increased willingness-to-pay for private care. SAC also played a key role in the burden of comorbidities such as asthma, nasal symptoms, and food allergies, which worsened the QoL. Reduced work productivity was reported by 44.78% of patients, with an average productivity loss of 15.72%. The analysis emphasizes the need for better management practices regarding both private and public healthcare costs.^[7]^ Furthermore, the lack of timely medical intervention often exacerbates the condition. Many patients resort to self-treatment or over-the-counter medications instead of seeking specialized care, as observed in Portugal, where only 19.4% of patients consulted an ophthalmologist at the onset of symptoms. This delayed or inadequate management can prolong symptoms and worsen QoL. Educational strategies and better access to effective treatments could significantly alleviate the burden of conjunctivitis.^[39]^ In summary, conjunctivitis poses a multifaceted challenge to QoL, affecting patients' physical, emotional, and social well-being. The recurrent nature of the condition necessitates comprehensive management strategies to minimize its impact on patients and their families.

### Keratoconus (KCN) and Quality of Life (QoL)

This subsection summarizes findings from 15 studies examining the impact of KCN on QoL, covering visual function, psychological well-being, comorbidities, and the effects of different treatments and disease severity on patients' daily living and social interactions.

KCN significantly impacts patients' QoL due to its progressive nature, visual impairments, and associated challenges. Beyond reduced visual acuity, KCN affects multiple aspects of daily living, including physical, emotional, and social domains. This subsection explores the multifaceted relationship between KCN and QoL, considering visual and nonvisual factors, the role of comorbidities, and the influence of treatment modalities.

#### Visual acuity and its correlation with QoL

Reduced visual acuity is a hallmark of KCN and a primary determinant of its impact on QoL. Studies have consistently demonstrated that worse visual acuity is associated with lower vision-related quality of life (VRQoL) scores across various validated instruments. For instance, Kymes et al found that patients with binocular vision worse than 20/40 experienced significant reductions in QoL across multiple domains, such as mental health, role difficulties, and social functioning, although general health and eye pain were less affected.^[9]^ Similarly, Pinto et al highlighted the importance of best-corrected visual acuity (BCVA) in the better-seeing eye, showing that worse BCVA is strongly correlated with lower scores on the Keratoconus Outcomes Research Questionnaire.^[41]^ These findings underscore the critical role of visual acuity in determining the overall QoL in patients with KCN.

#### Refractive error and its correlation with QoL

The irregular astigmatism characteristic of KCN poses challenges for refractive correction, which directly impacts QoL. While spectacles may suffice in the early stages, the progression of the disease often necessitates more advanced solutions, such as rigid gas-permeable (RGP) or scleral lenses.^[42,43]^ However, these lenses can introduce their own challenges, such as discomfort, inconvenience, and complications. Steinberg et al reported that even in early stage KCN with relatively good corrected distance visual acuity, patients experienced a decline in QoL, particularly regarding driving and due to ocular pain subscales.^[44]^ Fournie et al further emphasized the financial and practical challenges associated with RGP lenses, such as discomfort, cost, and lens loss, which significantly impact QoL.^[12]^

#### Contrast sensitivity and daily activities

Reduced contrast sensitivity, a prominent feature of KCN, exacerbates difficulties in daily activities such as driving, reading, and facial recognition. Although not explicitly addressed in all studies, research has shown that reduced contrast sensitivity is a well-documented consequence of KCN that possibly contributes to patients' difficulties in specific tasks. Studies such as those by Fan et al and Al Bdour et al have highlighted the importance of contrast sensitivity in determining functional outcomes and QoL in patients with KCN.^[11,13]^ Further research is warranted to better understand the relationship between contrast sensitivity and QoL outcomes in this population.

#### Ocular pain and discomfort

Ocular pain and discomfort are common complaints among patients with KCN, significantly contributing to reduced QoL. These symptoms may arise from the disease itself, contact lens wear, or surgical interventions. In early stage KCN, patients reported significantly lower scores on the NEI-VFQ subscale for ocular pain compared to emmetropes and myopes.^[44]^ Fournie et al also found discomfort to be a recurring theme in patient interviews, particularly among those using RGP lenses.^[12]^ Fan et al detailed additional symptoms such as dryness, irritation, and photophobia, emphasizing their negative impact on daily activities and emotional well-being.^[13]^

#### Comorbidities and QoL impact

Comorbidities such as VKC, Down syndrome, atopic dermatitis, and Leber congenital amaurosis often exacerbate the impact of KCN on QoL. VKC, for instance, increases ocular discomfort, itching, and photophobia.^[45]^ Similarly, conditions like atopic dermatitis introduce additional ocular surface challenges, while Leber congenital amaurosis compounds visual impairment. Although the interplay between these comorbidities and KCN is not always explicitly addressed, they undeniably contribute to a more complex disease burden and further reductions in QoL.

#### Impact on specific activities

KCN significantly affects activities such as driving, reading, and social interactions. Driving, particularly at night, is a major concern for patients with KCN. Steinberg et al reported lower scores on the NEI-VFQ driving subscale for patients with early stage KCN compared to controls.^[44]^ Reading and computer use are also impaired due to blurred and distorted vision, while social interactions may be impaired due to self-consciousness about eye appearance and functional limitations.^[12,13]^

#### QoL across KCN severity grades and populations

Disease severity plays a crucial role in determining QoL outcomes. Generally, more severe forms of KCN are associated with greater visual impairments and lower QoL scores. Kymes et al and Pinto et al demonstrated a clear correlation between worsening clinical parameters and lower QoL scores.^[9,41]^ However, some authors, such as Gothwal et al, have found no significant differences in QoL between moderate and severe KCN groups, possibly due to limitations of the questionnaires used.^[10]^ Variations in QoL outcomes across populations have also been observed and attributed to cultural, socioeconomic, and healthcare access factors. For example, the Saudi Arabian study found that females and younger patients reported lower QoL, while the Jordanian study noted lower visual function scores in males.^[11,46]^

In summary, KCN significantly impacts QoL through a combination of visual and nonvisual factors, including reduced visual acuity, irregular astigmatism, contrast sensitivity deficits, and ocular discomfort. Comorbidities and challenges in specific activities further compound the disease burden. While the impact on QoL is generally consistent across populations, variations due to demographic and cultural factors highlight the need for individualized approaches to KCN management. Understanding these multifaceted impacts is critical for optimizing treatment strategies and improving the overall well-being of patients with KCN.

### Keratitis and Quality of Life (QoL)

This subsection reviews the analysis of eight studies exploring how QoL is affected by various types of keratitis, including infectious keratitis (IK), microbial keratitis (MK), and Acanthamoeba keratitis (AK), and highlights the visual, psychological, social, and economic aspects of the disease.

Patients with keratitis, a condition that can threaten vision, experience a significant deterioration in QoL. Whether due to vision disturbances, lengthy treatment regimens, or the development of complications, keratitis severely affects patients' QoL. Patients with IK often experience low VRQoL, as assessed by the NEI VFQ-25. In a recent study conducted in China, patients with IK averaged a composite score of 58.1 on the NEI VFQ-25, with the lowest subscale scores associated with role difficulties and ocular pain. The BCVA in the worst-seeing eye, disease duration, prior surgery, and sex were significant predictors of VR-QoL. Early treatment was associated with better QoL outcomes, highlighting the importance of timely intervention to minimize the long-term impact of IK.^[47]^

#### Social life and occupation

In sub-Saharan Africa, MK has been described as a “silent epidemic” owing to its high prevalence and profound effects on QoL.^[48]^ A study conducted in Uganda revealed that MK causes a severe reduction in QoL during the acute phase, particularly in vision-specific domains such as visual symptoms (mean score 20.7) and general functioning (42.8). Even after treatment and resolution of symptoms, QoL scores among patients with MK remained lower than those of unaffected controls. Notably, visual acuity at 3 months and a history of eye loss were significant determinants of QoL. Surprisingly, individuals who underwent eye removal and received prostheses reported better QoL scores than those with persistent symptoms, likely due to the alleviation of pain and discomfort.^[49]^

AK is another severe form of keratitis that significantly compromises QoL. Patients with AK often endure prolonged treatment periods, frequent follow-up visits, and, in severe cases, surgical interventions such as penetrating keratoplasty.^[50]^ A delayed diagnosis of AK is associated with poorer outcomes, as the disease often progresses to deep stromal involvement, necessitating intensive and long-term care. The chronic pain, extended recovery timelines, and potential surgical complications further exacerbate the negative impact on QoL. Additionally, patients with AK face challenges in their personal and professional lives due to the disease's debilitating effects.^[51]^

In summary, across all types of keratitis, timely and accurate diagnosis is crucial to improving patient outcomes and minimizing the QoL burden. Early intervention can shorten treatment durations, reduce the need for invasive procedures, and enhance visual outcomes, ultimately improving patients' psychological and social well-being. By addressing the physical and emotional challenges posed by keratitis, healthcare providers can help mitigate its detrimental effects on QoL.

### Corneal Dystrophies and Quality of Life (QoL)

This subsection summarizes findings from five key studies investigating the impact of various corneal dystrophies on QoL, focusing on psychological well-being, visual function, symptom burden, and economic implications.

#### Psychological well-being

Corneal dystrophies significantly impact patients' QoL due to their progressive nature, visual impairment, and associated symptoms like pain and discomfort. A survey using the NEI VFQ-25 in Japanese patients with Fuchs endothelial corneal dystrophy (FECD) demonstrated lower composite scores (77.6 ± 11.0) compared to healthy controls (89.2 ± 7.0), with subscales for distance vision and mental health being the most affected. Despite the correlation between disease severity and QoL, corrected distance visual acuity of the dominant eye did not significantly correlate with composite scores, suggesting that other factors, such as mental and emotional health, play pivotal roles in QoL impairment.^[52]^

#### Economic burden

Corneal dystrophies such as epithelial-stromal TGFBI dystrophies and stromal dystrophies also cause substantial QoL reductions due to recurrent corneal erosions and progressive visual decline. Symptoms like glare, photophobia, and scarring are particularly debilitating, especially during advanced stages. Patients with endothelial dystrophies, such as FECD, experience significant visual and functional limitations that worsen as the disease progresses, often necessitating surgical interventions. The chronic nature of the diseases, frequent medical visits, and the potential need for repeated surgeries further exacerbate the psychological and physical burden on patients, underscoring the significance of timely diagnosis and effective management strategies.^[53]^

In summary, corneal dystrophies greatly impair QoL through gradual vision deterioration, discomfort, and emotional challenges, with mental health playing a key role beyond just vision sharpness. Symptoms such as glare and light sensitivity intensify as the disease advances, causing significant functional difficulties and frequently necessitating surgical intervention. The ongoing progression and repeated treatments contribute to psychological, physical, and financial strain, emphasizing the importance of early detection and proper management.

### Trichiasis and Quality of Life (QoL)

This subsection is based on four key studies examining the impact of trichiasis on QoL, focusing on physical symptoms, psychological well-being, and the benefits of surgical management. Trichiasis profoundly affects QoL due to its physical, psychological, and social consequences.

#### Psychological well-being

Studies reveal that trichiasis causes significant discomfort, including pain, photophobia, and watering, which interfere with daily activities and reduce physical functioning, even in individuals with normal vision. Patients with trichiasis report lower QoL scores in physical and psychological health domains compared to controls. However, social relationships are often unaffected, indicating no substantial stigma or exclusion associated with the condition.^[54]^

#### Effective management

Surgical intervention for trichiasis significantly improves QoL by alleviating pain and enabling better engagement in daily activities. A longitudinal study in Ethiopia showed that 1 year after trichiasis surgery, the proportion of patients performing daily tasks without difficulty increased substantially.^[55]^ This improvement was independent of visual acuity changes, highlighting the role of symptom relief in enhancing functional capabilities and reducing dependence on others. The study also reported reduced ocular pain and its interference with personal care, sleep, and productivity, further contributing to QoL improvement.^[55]^

Moreover, untreated trichiasis is associated with psychological distress, including anxiety and fear of vision loss. Timely surgical management not only prevents blindness but also alleviates these emotional burdens, promoting overall well-being and social reintegration.^[55]^ Addressing barriers to accessing treatment is essential to reduce the physical and psychological suffering caused by trichiasis.^[54,55]^ A case–control study further emphasized that trichiasis negatively impacts QoL even without visual impairment, underscoring the need for early intervention to mitigate its broader effects.^[56]^

In summary, trichiasis significantly impairs QoL through physical discomfort and psychological distress, even without vision loss. Surgical treatment effectively relieves symptoms, improves daily functioning, and reduces emotional burdens. Early intervention and overcoming treatment barriers are essential to prevent blindness and enhance overall well-being and social reintegration.

### Glaucoma and Quality of Life (QoL)

This section summarizes findings from seven key studies examining the multifaceted impact of glaucoma on QoL.

Glaucoma significantly impacts QoL across multiple dimensions, including physical, psychological, and social domains. As a chronic, progressive eye disease, it not only causes irreversible damage to vision but also affects patients' emotional well-being, independence, and overall functionality.^[57,58,59,60,61,62,63]^ This section explores the factors influencing QoL in patients with glaucoma, the role of disease severity, and the sociodemographic and psychological correlates of QoL.

#### Physical and functional limitations

The physical impact of glaucoma is primarily driven by visual impairment, which affects patients' ability to perform daily activities such as reading, driving, and navigating unfamiliar environments. Studies have consistently shown that QoL scores decrease with worsening disease severity, especially as visual field loss progresses. Patients with advanced primary open-angle glaucoma report significantly reduced health utility scores compared to those with early stages of the disease, highlighting a critical threshold where QoL deteriorates substantially.^[59]^ Visual acuity in the better eye plays a significant role in determining QoL, as patients often rely on this eye for functional vision.^[57]^ Furthermore, patients with lower vision in their better eye report greater challenges in mobility, reading, and maintaining independence.^[58]^

Peripheral vision loss, a hallmark of glaucoma, has been associated with difficulties in performing outdoor activities and increased risks of accidents. For instance, patients with advanced glaucoma often experience challenges with activities requiring peripheral vision, such as driving, which significantly contributes to reduced independence and QoL impairment.^[59]^ However, the relationship between visual impairment and QoL is not always linear: individuals with moderate disease may still maintain relatively good QoL due to adaptive mechanisms.^[60]^ This underscores the resilience of some patients in coping with functional limitations despite disease progression.

#### Psychological and emotional challenges

Glaucoma is closely associated with psychological distress, including anxiety, depression, and fear of blindness. The chronic and irreversible nature of the disease often leads to heightened emotional strain, particularly in patients with advanced stages of glaucoma. Younger individuals and those with high intraocular pressure (IOP) have reported significantly higher levels of psychological distress due to the knowledge of potential disease progression.^[57]^ Anxiety and depression are prevalent in patients with glaucoma, as they face uncertainties regarding their future vision and independence.^[58]^

The psychological burden is further amplified by the need for lifelong treatment and frequent clinical visits. Patients often experience frustration with the complexity of medication regimens, side effects, and the perceived inefficacy of treatment.^[59]^ Women, in particular, have been found to have lower QoL scores across all domains, reflecting gender disparities in healthcare access, support systems, and societal expectations.^[57]^

Despite these challenges, some patients exhibit emotional resilience and accept their condition over time. Adaptive coping mechanisms, such as reliance on social support, humor, and optimism, have been shown to mitigate the psychological impact of glaucoma. However, maladaptive strategies, such as avoidance and nonadherence to treatment, are more common among younger patients and those with no significant visual impairment, potentially increasing the risk of disease progression.^[61]^

#### Sociodemographic and economic factors

Sociodemographic variables, including age, gender, marital status, income, and literacy, play a significant role in shaping the QoL of patients with glaucoma. Women, particularly those from lower socioeconomic backgrounds, report poorer QoL due to limited access to resources, greater caregiving responsibilities, and societal barriers.^[57]^ Income disparities have also been shown to affect QoL, as patients from below the poverty line often face delays in diagnosis and treatment due to the high cost of glaucoma management.^[57]^ This economic burden contributes to psychological distress and reduced adherence to treatment.^[59]^

Marital status and social support are protective factors for QoL. Married individuals often report better QoL due to the availability of emotional and practical support from their partners. Conversely, single or divorced patients may experience unmet social needs, further exacerbating their psychological burden.^[57]^ Literacy and education levels have also been associated with better QoL, as they enable patients to understand their condition and adhere to treatment regimens effectively.^[57]^

In summary, QoL in patients with glaucoma is influenced by a complex interplay of physical, psychological, and sociodemographic factors. While disease severity and visual impairment remain the primary determinants, psychological distress and socioeconomic disparities also play critical roles. Comprehensive glaucoma management should address not only the medical aspects of the disease but also the emotional and social needs of patients. Early intervention, patient education, and tailored support systems can improve adherence to treatment and enhance QoL for individuals living with glaucoma. Future research should focus on developing glaucoma-specific tools to better capture the unique challenges faced by patients and guide individualized care strategies.

### Cataract and Quality of Life (QoL)

This part reviews the results of 13 studies examining the impact of cataract and cataract surgery on QoL.

Cataracts significantly affect QoL through their impact on vision-related functioning, independence, and mental well-being.^[34,64,65,66,67]^ Cataract surgery, as one of the most effective treatments, has demonstrated notable improvements in both vision-specific and general QoL measures, though the extent of improvement varies depending on individual circumstances.^[33,35,36,38,68,69,70,71]^

#### Psychological well-being

Studies consistently highlight the negative association between cataract symptoms and QoL. Lee et al found that symptoms such as blurry vision and glare were highly correlated with reduced VRQoL, as measured by tools such as the VF-14 and the Global Measure of Vision. These symptoms often interfere with daily activities, reducing independence and satisfaction with vision.^[66]^ Similarly, Oleś et al reported that visual impairment caused by cataracts led to diminished health-related QoL (HRQoL) and increased dependence on others, particularly in older adults. The severity of symptoms was linked to greater declines in autonomy and self-esteem, further compounding the negative impact on QoL.^[35]^

#### Effective management

Cataract surgery has been shown to yield substantial improvements in VRQoL. Uusitalo et al demonstrated that the VF-7, a visual-functioning index, captured substantial gains in patient satisfaction and functional ability following cataract surgery.^[33]^ These improvements were seen in areas such as reading, mobility, and household tasks. Groessl et al also observed significant enhancements in QoL after cataract surgery, particularly in vision-specific domains measured by the NEI-VFQ25. The study noted that most improvements occurred within the first month post-surgery, with some additional gains persisting over 6 months.^[68]^

However, the relationship between cataract surgery and general QoL measures is less straightforward. Oleś et al noted that while patients experienced significant improvements in HRQoL post-surgery, general QoL indices did not show comparable gains.^[35]^ This discrepancy may be attributed to factors such as unmet expectations, socioeconomic barriers, or adaptation challenges. Similarly, Groessl et al found that generic HRQoL measures such as the SF-36v2TM were less sensitive to changes post-surgery, emphasizing the importance of using vision-specific tools to assess the outcomes.^[68]^

The benefits of cataract surgery extend beyond vision improvement. Musch et al reported that cataract extraction in patients with glaucoma not only improved visual acuity but also enhanced vision-specific QoL, as measured by the Visual Activities Questionnaire. The study observed significant reductions in glare disability, improved depth perception, and better visual processing speed after surgery.^[65]^ These findings align with those of Hahn and Krummenauer, who highlighted the cost-effectiveness of cataract surgery in terms of quality-adjusted life years, reinforcing its broader societal and economic value.^[72]^

Cataract surgery for the second eye has also been associated with additional QoL benefits. Lundström et al found that patients who underwent bilateral cataract surgery reported greater satisfaction and functional improvements than those who had surgery in only one eye. Enhanced stereoacuity and reduced visual interference from the untreated eye were key contributors to these outcomes.^[71]^

Despite these positive outcomes, disparities in access to surgery and variations in postoperative care can limit improvements in QoL. Oleś et al emphasized that patients from lower socioeconomic backgrounds often face greater challenges in realizing the full benefits of cataract surgery, including limited access to follow-up care and rehabilitation.^[35]^ Similarly, Lee et al noted cultural differences in the timing of surgery and patients' perceptions of its necessity, which may influence QoL outcomes.^[66]^

#### Impact of age and comorbidities on QoL

Age and comorbidities also play a role in determining QoL outcomes. Musch et al found that older patients and those with diabetes or pseudoexfoliative glaucoma had higher risks of cataract progression and surgery, but also experienced significant QoL gains post-surgery. However, the presence of advanced glaucoma may limit the extent of these improvements, particularly in terms of visual field measures.^[65]^

In summary, cataracts have a profound impact on QoL, affecting both functional and emotional well-being. Cataract surgery remains a highly effective intervention, significantly improving vision-specific QoL and, to a lesser extent, general QoL. However, disparities in access, cultural perceptions, and individual comorbidities can influence these outcomes. Future research should focus on addressing these barriers and optimizing postoperative care to maximize QoL improvements for all patients.

##  CONCLUSION

Anterior segment diseases significantly impact patients' QoL by affecting their physical health, mental well-being, social interactions, and economic productivity. Conditions such as DED, KCN, keratitis, and others pose multifaceted challenges that extend beyond vision impairment to encompass chronic discomfort, emotional distress, and functional limitations in daily activities.

Effective management strategies must address both clinical and psychosocial aspects to improve patient outcomes. Early diagnosis, targeted interventions, and the use of validated QoL assessment tools are essential for understanding the broader impact of these diseases and guiding patient-centered care. Future research should focus on developing innovative treatment approaches and multidisciplinary care models that enhance not only ocular function but also the overall well-being of affected individuals.

##  AI Use Disclosure

Artificial intelligence applications were utilized for English language editing to improve clarity, grammar, and overall readability. However, the authors conducted all scientific content and interpretations without relying on AI tools.

##  Financial Support and Sponsorship

None.

##  Conflicts of Interest

None.
